# CD133 and CD166 Stem Cells Markers Expression, Clinicopathological Parameters, and Fragmentation Response Patterns of ypT3 Rectal Cancer Following Neoadjuvant Chemoradiotherapy

**DOI:** 10.3390/biomedicines13061300

**Published:** 2025-05-26

**Authors:** Diana Lavinia Pricope, Adriana Grigoraș, Gabriel Mihail Dimofte, Cristina Terinte, Cornelia Amalinei

**Affiliations:** 1 Department of Morphofunctional Sciences I, Grigore T. Popa University of Medicine and Pharmacy, 700115 Iasi, Romania; dianalavinia64@gmail.com; 2 Department of Histopathology, Institute of Legal Medicine, 700455 Iasi, Romania; 3 Surgical Department, Grigore T. Popa University of Medicine and Pharmacy, 700115 Iasi, Romania; gdimofte@gmail.com; 4 2nd Clinic of Surgical Oncology, Regional Institute of Oncology, 700483 Iasi, Romania; 5 Pathology Department, Regional Institute of Oncology, 700483 Iasi, Romania; cterinte@gmail.com

**Keywords:** rectal cancer, ypT3 stage, neoadjuvant chemoradiotherapy, fragmentation pattern, CD133, CD166, immunohistochemistry, LARC

## Abstract

**Background**: The effectiveness of neoadjuvant chemoradiotherapy (nCRT) is variable in locally advanced rectal cancer (LARC) patients, the ypT3 stage having a minimal or moderate response. The aim of our study was the evaluation of the association between CD133 (Prominin1) and CD166 (ALCAM) expression, survival parameters, and clinicopathological characteristics of a subgroup of LARC patients who achieved ypT3, showing post-nCRT and TME tumor fragmentation response and the assessment of these CSCs biomarkers value as indicators of the nCRT tumor response. **Methods**: Our study group comprised 60 LARC patients who achieved ypT3 status and exhibited a tumor fragmentation pattern following nCRT. Clinicopathological parameter and survival evaluations, along with CD133 and CD166 immunohistochemistry and scoring, were performed and the associations between different parameters were tested. **Results**: High CD133 expression was significantly associated with ypN category (*p* = 0.018), lymphovascular invasion (LVI) (*p* = 0.009), perineural invasion (PnI) (*p* = 0.006), and tumor grading (*p* = 0.047), while high CD166 expression was significantly associated with LVI (*p* = 0.020) and PnI (*p* = 0.028). Tumors with high CD133 and CD166 expressions were associated with decreased overall survival (OS) (*p* = 0.004 and *p* = 0.006). Cox regression analysis identified high CD133 and CD166 expression as independent factors associated with reduced survival (HR = 3.237, *p* = 0.014 and HR = 2.866, *p* = 0.020). **Conclusions**: Our results support the hypothesis that CD133 and CD166 are putative CSC biomarkers associated with aggressive behavior and a poor prognosis in LARC, offering opportunities for personalized targeted therapies.

## 1. Introduction

Despite the recent advances in oncological therapy, colorectal cancer (CRC) is still considered one of the most common malignancies, being the second-leading cause of cancer-related mortality worldwide [1,2]. CRC accounted for 915,880 deaths worldwide in 2020 [3], with 1.6 million estimated deaths by 2040 [4]. Moreover, CRC-related fatalities represented 10.6% of all cancer-related deaths in Europe in 2020 [3].

Although not all mechanisms involved in CRC carcinogenesis have been elucidated, several risk factors have been recognized, including a sedentary lifestyle, a high-fat diet, smoking and alcohol abuse, along with genetic predisposition [5].

More than 30% of CRCs have a rectal location, with a median age at diagnosis of 70 years [2]. While it is acknowledged that colon and rectal cancer (RC) share similar histological features, it is generally accepted that RC represents a distinct entity due to significant differences regarding therapy, prognosis and biological features [5,6]. About 60% of RCs are diagnosed in the locally advanced stage (LARC), which may only benefit from neoadjuvant chemoradiotherapy (nCRT), followed by total mesorectal excision (TME), as a common therapeutic approach [7,8,9].

The histopathological evaluation of TME specimens provides crucial details regarding the LARC prognosis, such as the American Joint Committee on Cancer (AJCC) tumor regression grade (TRG) and ypTN staging [9,10]. Several studies have demonstrated that the TRG and ypTN staging are not directly correlated, leading to different categories of patients [10,11]. Thus, a T3N+ LARC patient who histologically achieves ypT3N0 and TRG1 (moderate post-nCRT therapeutic response) has a lower predictive risk of progression compared to a ypT3N0 TRG3 patient with a minimal post-nCRT therapeutic response [10]. However, the lack of correlation between these parameters could be attributed to TRG classification variability and to the inconsistent description of the therapy response pattern in comparison with the tumor regression grade [9,12].

RC tumor regression patterns are characterized by tumor shrinkage or fragmentation in response to nCRT [10,13,14]. While shrinking represents the centrifugal tumor’s reduction towards the lumen, tumor fragmentation is defined as the disintegration of the tumor mass into multiple clusters and fragments of variable shapes and sizes, which may be extended as much as the initial tumor limits [12]. This response type is achieved in 40–80% of LARC patients and is more frequently associated with lymph node metastases and positive surgical margins [14]. The fragmentation response pattern is often correlated with an advanced ypT stage and poor prognosis, considering that tumor fragmentation significantly increases the probability of cancer cells’ deep invasion of the bowel layers [13,14]. Intriguingly, due to the small size of the fragments, being below the imaging technique’s resolution, the surgical intervention may be incomplete, possibly resulting in local recurrence [11]. The majority of LARC patients who do not respond to nCRT maintain a ypT3-stage diagnosis, which is characterized by the presence of residual malignant cells beyond the *muscularis propria* layer, within the perirectal tissue or the mesorectum [15].

However, post-nCRT residual tumor transgression into all rectal wall layers up to the mesorectum was associated with a minimal impact on tumor recurrence and overall survival in ypT3 patients whounderwent TME surgery and achieved negative circumferential resection margins (CRMs) [15,16].

As a consequence of tumor heterogeneity, only a limited proportion of LARC patients show a positive response to nCRT regimens, leading to numerous studies aiming to identify tumor radio-resistance mechanisms [2,5,17,18]. In this context, recent studies have demonstrated that the CRT response is mediated by cancer stem cells (CSCs), expressing ALDH1, Sox2, Oct-4, Lgr5, CD24, CD26, CD29, CD44, CD51, CD133, CD166, Nanog and EpCAM surface markers, which are a subpopulation of RC tumor cells distinguished by their stemness properties and self-renewal capacities [2,19,20,21]. CSCs’ characteristics make them significant drivers of tumor growth and recurrence, as well as significant factors in prognosis and management [22,23].

Among the characteristic panel of markers, CD133, known as Prominin1 or PROM1, is considered the most common CSC biomarker [7]. CD133 has been isolated in numerous solid tumors, including prostate, lung, brain and liver cancers, being also useful for CRC CSC identification [7,24,25]. Studies focused on the significance of CD133 expression in RC have demonstrated that CD133-positive cells exhibit stronger resistance to chemoradiotherapy protocols and a risk of cancer recurrence compared to CD133-negative cells [7,19,26].

Another stem cell marker is CD166 (ALCAM), a type-1 glycoprotein belonging to the immunoglobulin superfamily, recognized for its role in the maintenance of CSCs’ characteristics, such as tumor initiation, growth and invasion [27]. This glycoprotein has been isolated in numerous solid tumors, but its significance as a potential prognostic marker has been particularly highlighted in CRC and esophageal squamous cell carcinoma [27,28]. This is attributed to the correlation between its overexpression and its aggressive clinicopathological features, in addition to reduced survival in RC and CRC [27,29,30].

Taking into consideration the significance of CRC CSCs, our study aimed at the identification of the clinicopathological characteristics of a subgroup of LARC patients who achieved ypT3, showing a post-nCRT and TME tumor fragmentation response, as well as the evaluation of the association between CD133 and CD166 expression, survival parameters and clinicopathological characteristics and the assessment of the CSC biomarkers as valuable indicators of the nCRT tumor response.

## 2. Materials and Methods

### 2.1. Patients and Tissue Samples

Our study group was selected from the LARC patients registered in the files of the Pathology Laboratory of the Regional Institute of Oncology Iasi, over a period of seven years (between 2017 and 2023). Out of a total of 238 LARC patients with variable ypT (ypT0–ypT4), 60 cases of ypT3 were selected. The inclusion criteria applied for case selection were the following: a minimum age of 18 years; the administration of neoadjuvant therapy [radiotherapy combined with concurrent chemotherapy with CAP (capecitabine), CAPEOX (capecitabine and oxaliplatin) or FOLFOX (leucovorin calcium, fluorouracil and oxaliplatin)], followed by TME; a ypT3-stage diagnosis; and a tumor fragmentation response following therapy. The exclusion criteria applied during case selection were the following: RC patients who underwent surgical intervention without prior neoadjuvant therapy; RC patients who had a “watch and wait” approach; and patients who received neoadjuvant treatment but refused surgical resection. Following the application of these criteria, 60 patients who achieved the ypT3 stage and exhibited a tumor fragmentation pattern after nCRT followed by TME were selected. Fragmentation was defined as clusters of cells that did not form a tumor mass, located at a distance of at least 3 mm from the main tumor mass [12].

All selected cases were reclassified following the criteria defined in the last WHO classification [31]. Following reclassification, the tumor regression grade (TRG) after neoadjuvant therapy was evaluated using the Dworak tumor response grading system (Table 1) [32,33].

Tumor budding (Bd) was evaluated according to the guidelines of the International Tumor Budding Consensus Conference (ITBCC), held in 2016 [34], as the presence of individual tumor cells or small clusters (up to a maximum of four cells) at the invasive tumor front (ITF). According to the ITBCC recommendations, Bd was evaluated as follows: 0–4 cells as Bd1, 5–9 cells as Bd2 and ≥10 cells as Bd3.

Lymphovascular invasion (LVI), registered in cases with tumor cell identification within the small endothelium-lined lymphatic or blood vessels, and perineural invasion (PnI), registered in cases with cancer cell invasion around, in and through nerves or nerve sheaths, were recorded [35]. Extramural vascular invasion (EMVI) and intramural vascular invasion (IMVI) were also registered according to the current recommendations [31].

Furthermore, poorly differentiated clusters (PDCs), defined as multiple groups of tumor cells, each consisting of at least five cells located at the ITF [34,36], were assessed according to the same stages and scoring classes used in the Bd evaluation, resulting in the following grades: PDC 1 with 0–4 PDCs, PDC 2 with 5–9 PDCs and PDC 3 with ≥10 PDCs [36].

Overall survival (OS) was registered as the period of time between the diagnosis and the date of death or of the last follow-up [35], considering that the latest was 31 March 2023.

The informed consent of the patients was obtained, and ethical approval was granted by the Ethics Committee of the Regional Institute of Oncology Iasi (no. 1267/4 July 2022) and by the Ethics and Research Committee of “Grigore T. Popa” University of Medicine and Pharmacy, Iasi (no. 249/19 December 2022).

### 2.2. Immunohistochemical Method

Based on microscopic examination, a representative formalin-fixed paraffin-embedded block of tissue for the diagnosed T stage, TRG, Bd and PDC grades, LVI and PnI status was selected for the immunohistochemical method for each case included in our study group, and consecutive 4-µm-thick sections were obtained.

The method involved the sections’ deparaffinization in xylene (for 40 min at 58 °C, followed by 10 min at room temperature), rehydration through successive baths in decreasing alcohol concentrations (100%, 90%, 80% and 70%, respectively) and antigenic epitope unmasking by the heat-induced epitope retrieval (HIER) method, with a pH 9 epitope retrieval solution (Leica Biosystems). The endogenous peroxidase was blocked with 200 μL 3% hydrogen peroxide for 10 min. Incubation with anti-CD133 (rabbit polyclonal antibody, ab19898, dilution 1/200, Abcam, Cambridge, MA, USA) and anti-CD166 (rabbit polyclonal antibody, ab109215, dilution 1/100, Abcam, Cambridge, MA, USA) primary antibodies was performed overnight at 4 °C. The reaction was developed using a compatible detection system (Abcam, Cambridge, MA, USA, ab64261) and 3,3′-diaminobenzidine tetrahydrochloride (DAB) solution for five minutes, at room temperature, with Mayer’s hematoxylin counterstain for three minutes, followed by dehydration and mounting [37]. Positive and negative controls were performed according to the producers’ recommendations.

### 2.3. Evaluation of CD133 and CD166 Immunoexpression

Following the slides’ examination at 200× magnification for the evaluation of the patterns and the extent of CD133 and CD166 immunoexpression by two experienced pathologists (C.A. and A.G.), the current CD133 and CD166 scoring systems [27,38] were applied, while the differences were addressed through consensus.

The qualitative evaluation of CD133 immunopositivity considered the membrane staining of the apical surfaces of the tumor glands, along with the staining of the cellular debris within the tumor glands and their cells’ cytoplasm. Based on the percentage of positive cells assessed on the entire tumor, the investigated cases were classified into two groups as follows: tumors with <50% positive cancer cells and tumors with ≥50% positive cancer cells. Using the staining pattern and its extent, CD133 immunoreaction was scored from 0 to 4, leading to the following two scoring categories: low CD133 expression, including scores of 0–2, and high CD133 expression, including scores of 3 and 4 (Table 2) [38].

CD166 expression was considered positive if the staining showed a predominant membrane pattern, along with partial staining of the cancer cell cytoplasm [27]. The immunohistochemistry intensity was divided into four groups (Table 3). The percentage of positive cells was evaluated between 0 and 100%. The final score was obtained by multiplying the percentage (0–100%) by the staining intensity (0–3), resulting in scores ranging from 0 to 300, according to the literature data [27]. Considering the score distribution, the cut-off value was calculated by summing the median and the interquartile ranges. The score of 120 was considered as a cut-off to divide the tumors into low and high CD166 expression (Table 3).

### 2.4. Statistical Analysis

The statistical analysis was performed with the SPSS version 25 (IBM, Armonk, NY, USA) and Microsoft Excel 2016 (Microsoft, Redmond, WA, USA) programs. Continuous variable types are reported as means ± standard deviations (SDs). Correlations between clinical, demographical and histopathological parameters and CD133 and CD166 expression were determined by Pearson’s chi-squared test. The survival analysis was performed using Kaplan–Meier curves. The log-rank test (Mantel–Cox) was used to analyze the survival data. A Cox regression analysis was also performed to evaluate the prognostic significance of the immunohistochemical parameters. A *p*-value < 0.05 was established as statistically significant.

## 3. Results

### 3.1. Clinicopathological Characteristics

Out of the total number of LARC patients in the study group diagnosed with the ypT3 stage and exhibiting a tumor fragmentation pattern after nCRT and TME, 47 (78.3%) were men and 13 (21.7%) were women. The mean patients’ age at diagnosis was 63.93 ± 8.671 years (ranging from 45 to 82 years), with similar ages for both genders: 63.54 ± 8.771 years for women and 64.04 ± 8.735 years for men.

The most common histological tumor type was adenocarcinoma not otherwise specified (NOS) (54 cases; 90%), while mucinous adenocarcinoma, with a ≥50% mucinous component, was less common (six cases; 10%).

The most common clinical tumor stage (cT) was cT3, registered in 44 patients (73.3%), while the most common clinical N stage was N2, registered in 43 patients (71.7%). The postoperative ypN category indicated a favorable response to nCRT, with the majority of patients classified as ypN0 (32 patients; 53.3%), while a significant number of patients (21 patients; 35%) were diagnosed as ypN1. Tumor grading was low in 47 cases (78.3%) and high in 13 cases (21.7%).

**Figure 1:**
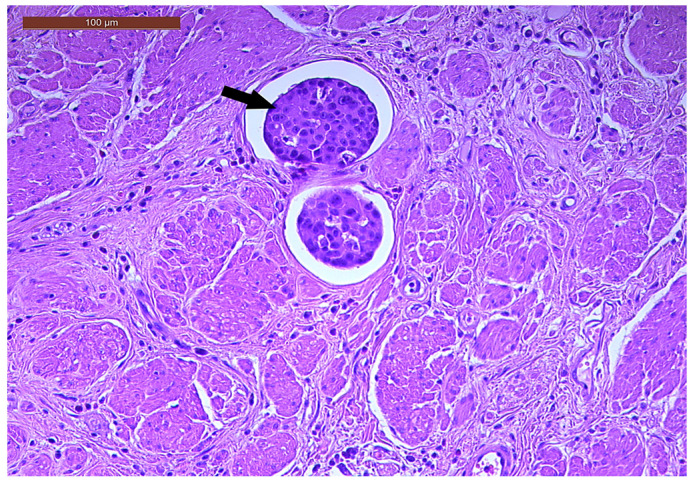
Lymphovascular invasion (arrow) in a ypT3-stage LARC case (H&E staining, 200×).

**Figure 2:**
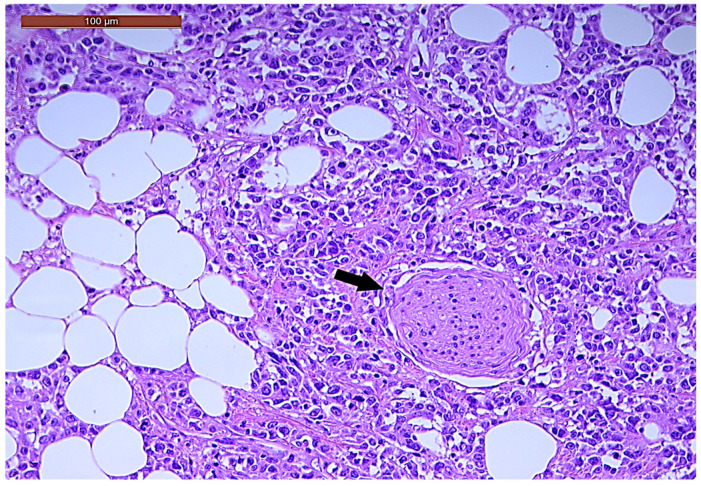
Perineural invasion (arrow) in a ypT3-stage LARC case (H&E staining, 200×).

**Figure 3:**
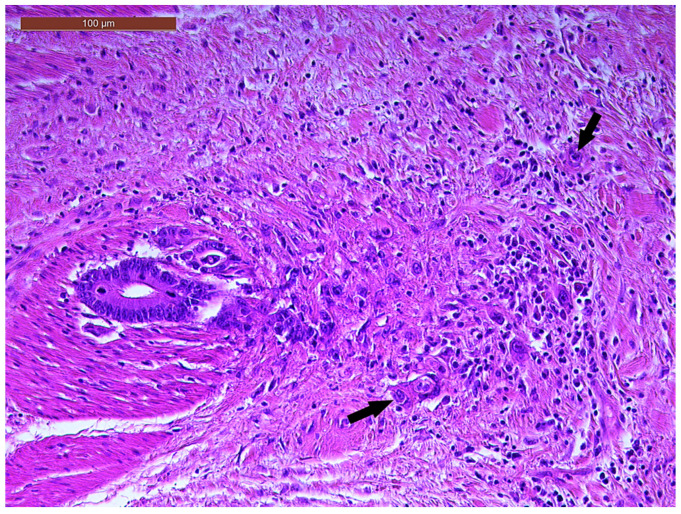
Individual and small clusters of tumor cells (arrows) at the invasive tumor front in a ypT3-stage LARC case (Bd2) (H&E staining, 200×).

**Figure 4:**
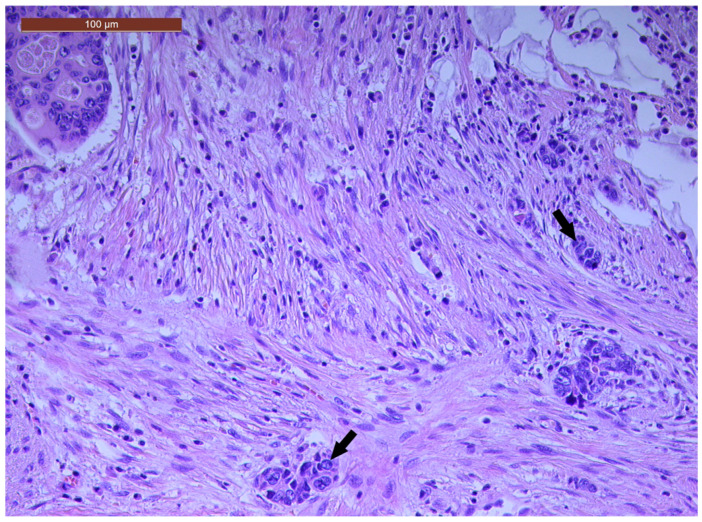
A few groups of tumor cells (arrows), consisting of at least five cells, located at the invasive tumor front in a ypT3-stage LARC case (PDC1) (H&E staining, 200×).

LVI was detected in 31 cases (51.7%), and perineural invasion (PnI) was identified in 20 cases (33.3%) (Figure 1 and Figure 2). EMVI and IMVI were registered in 31 (51.7%) and 21 cases (35%), respectively. Regarding Bd and PDC categories, a significant proportion of tumors were recorded as Bd1, while Bd2 was less common (49 cases; 81.6% vs. 7 cases; 11.7%) (Figure 3); moreover, a large proportion were classed as the PDC1 grade (Figure 4), while the PDC2 grade was less common (55 cases; 91.7% vs. 4 cases; 6.7%). The main clinicopathological characteristics of the study group are summarized in Table 4.

In total, 37 patients (61.7%) were survivors, while 23 patients (38.4%) were non-survivors, at the time of the patients’ enrolment in the study group (Table 4). The mean overall survival (OS) period of the study group was 37.48 ± 18.06 months (ranging from 7 to 91 months), with a median of 32 months. The mean OS in the study group was 36.15 ± 22.63 months for women and 37.85 ± 16.86 for men.

### 3.2. Qualitative and Semi-Quantitative Assessment of CD133 and CD166 Immunoexpression

**Figure 5:**
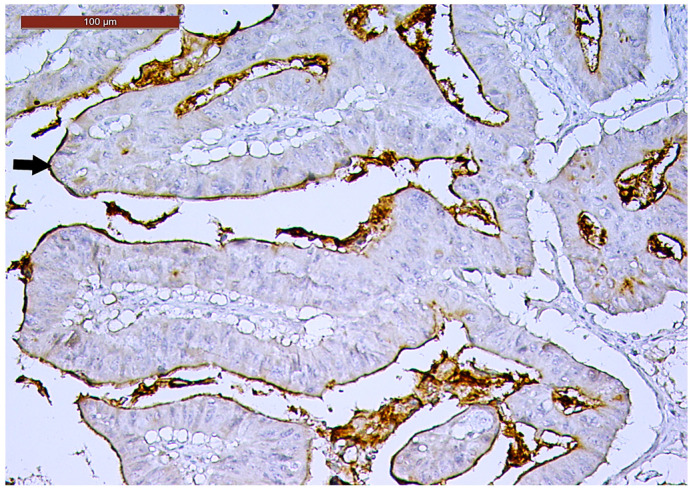
Luminal membrane CD133-positive immunoexpression (arrow) in more than 50% of the tumor area in a ypT3-stage LARC case (200×).

**Figure 6:**
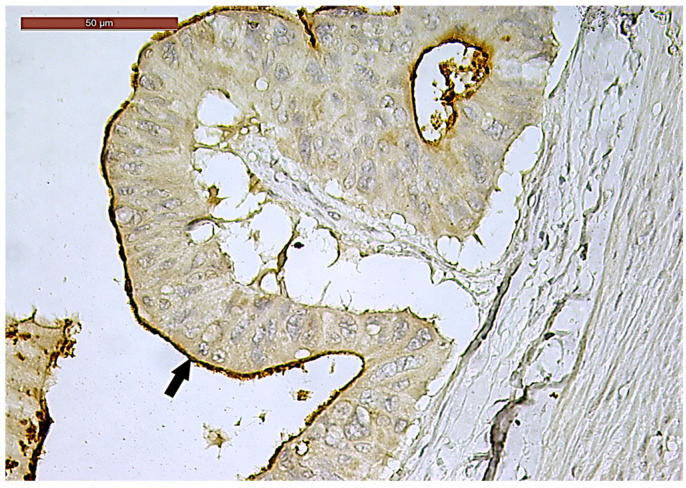
Luminal membrane (arrow) and cytoplasmic CD133-positive immunoexpression in more than 50% of the tumor area in a ypT3-stage LARC case (400×).

**Figure 7:**
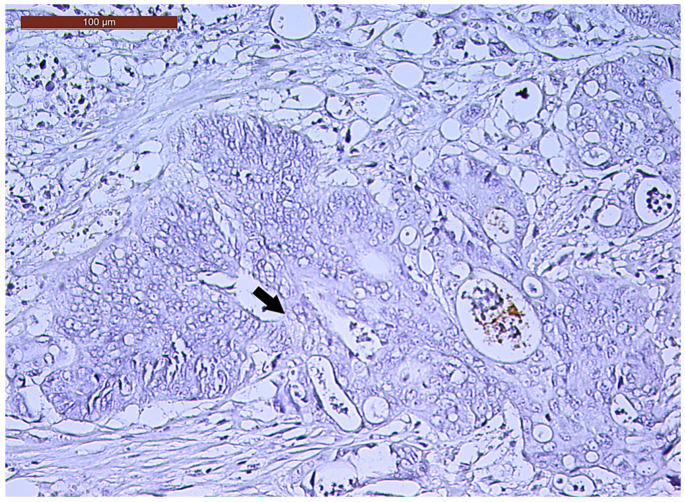
Negative CD133 immunoexpression (arrow) of a ypT3-stage LARC case (400×).

Regarding the qualitative and semi-quantitative evaluation of the CD133 immunoreaction pattern, different types of subcellular immunostaining localization were detected, as follows: exclusively membrane and luminal staining in less than 50% of the entire tumor area in 22 cases (36.6%), luminal membrane and cytoplasmic staining in less than 50% of the tumor area in nine cases (15%), only luminal membrane staining in more than 50% of the tumor area in 11 cases (18.3%) and luminal membrane and cytoplasmic staining in more than 50% of the tumor area in 16 cases (26.6%) (Figure 5 and Figure 6). CD133 expression was negative in two cases (3.3%) (Figure 7).

Following the scoring system applied in relation to the staining extent as less or more than 50% of the amount of cancer cells, low CD133 expression was observed in 33 cases (55%), while high CD133 expression was detected in 27 cases (45%).

**Figure 8:**
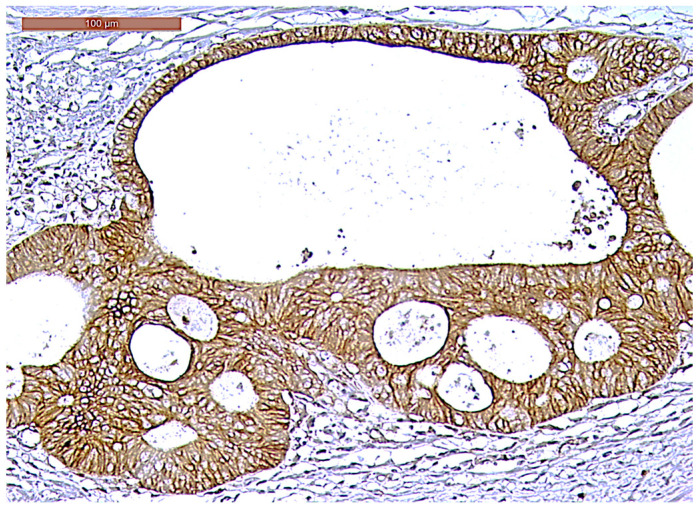
Strong CD166 immunoexpression of a ypT3-stage LARC case (200×).

**Figure 9:**
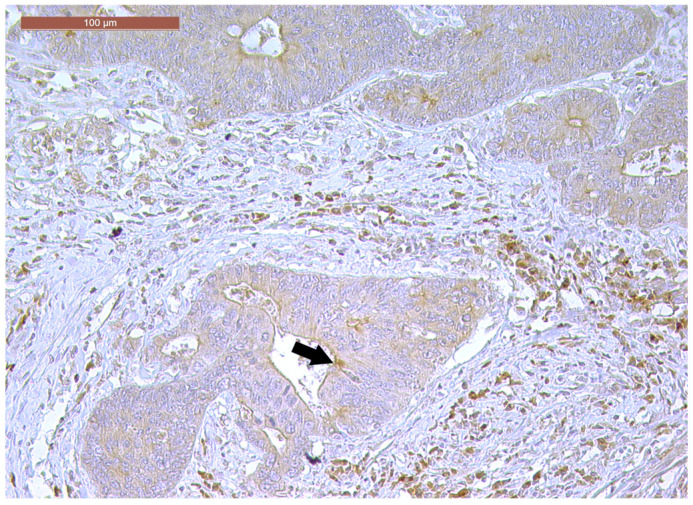
Moderately positive CD166 immunoexpression of a ypT3-stage LARC case (100×).

**Figure 10:**
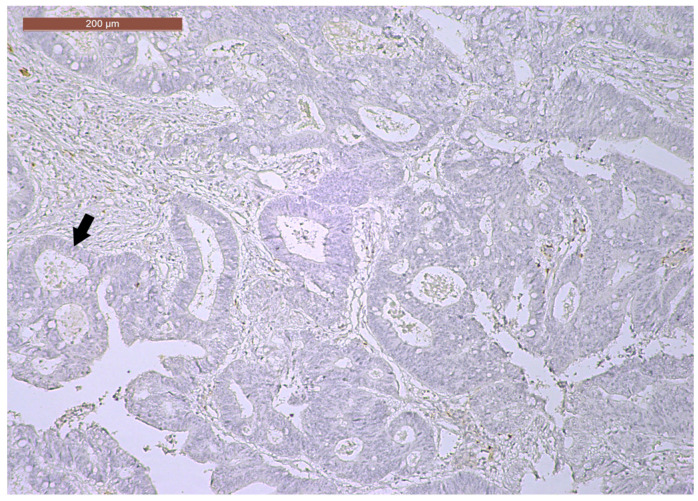
Weak CD166 immunoexpression of a ypT3-stage LARC case (40×).

CD166 expression was considered positive if showing predominant membrane location, with or without cytoplasmic expression. In terms of intensity, strong (3+) CD166 staining was observed in 14 cases (23.3%), while moderate (2+) and weak (1+) CD166 staining was found in 30 cases (50%) and 16 cases (26.7%), respectively. CD166 negative expression was not registered in any tumor (Figure 8, Figure 9 and Figure 10). According to the scoring system applied in the investigated cases, low CD166 expression was detected in 30 samples (50%), while high CD166 expression was registered in the other 30 samples (50%).

### 3.3. Correlation Between CD133 and CD166 Immunoexpression and Clinicopathological Characteristics

According to the scoring system, samples with high CD133 expression were significantly associated with LVI, PnI, ypN and the tumor grading (*p* = 0.009, *p* = 0.006, *p* = 0.018 and *p* = 0.047, respectively).

The statistical analysis of the association between the CD166 score and the clinicopathological characteristics revealed that a high CD166 score was significantly associated with PnI and LVI (*p* = 0.028 and *p* = 0.020, respectively).

The associations between the clinicopathological characteristics and CD133 and CD166 expression are summarized in Table 5.

### 3.4. Correlation Between CD133 and CD166 Expression and Survival

The analysis of patients’ survival in association with the CD133 score revealed a mean OS of 37.91 ± 17.18 months for patients with low CD133 tumor expression and 36.96 ± 19.40 months for patients with high CD133 tumor expression. In an analogous manner, the mean OS was 41.10 ± 19.82 months for patients with low CD166 tumor expression and 33.87 ± 15.61 months for patients with high CD166 tumor expression. According to the association of both CRC CSC markers’ expression, the mean OS for patients with low CD133 and CD166 tumor expression was 39.43 ± 18.40 months, and it was 35.33 ± 17.42 months for patients with high CD133 and CD166 tumor expression.

**Figure 11:**
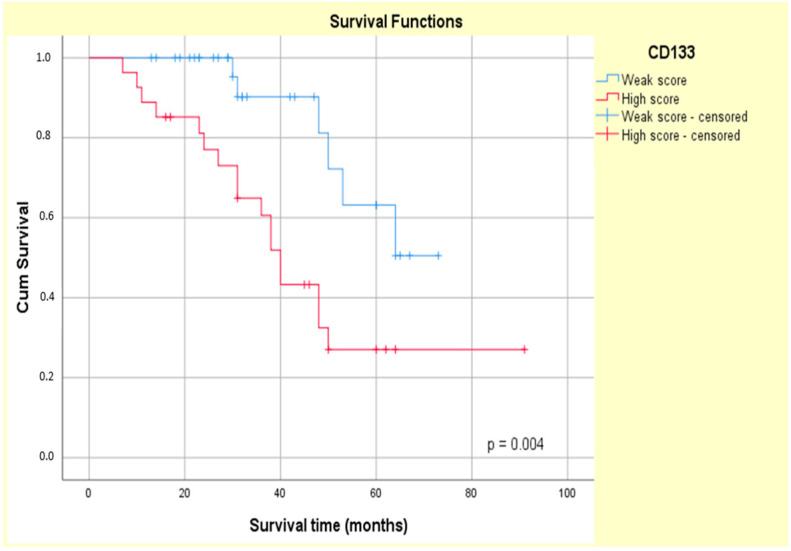
Kaplan–Meier survival curves for OS of ypT3-stage LARC patients with low or high CD133 expression.

**Figure 12:**
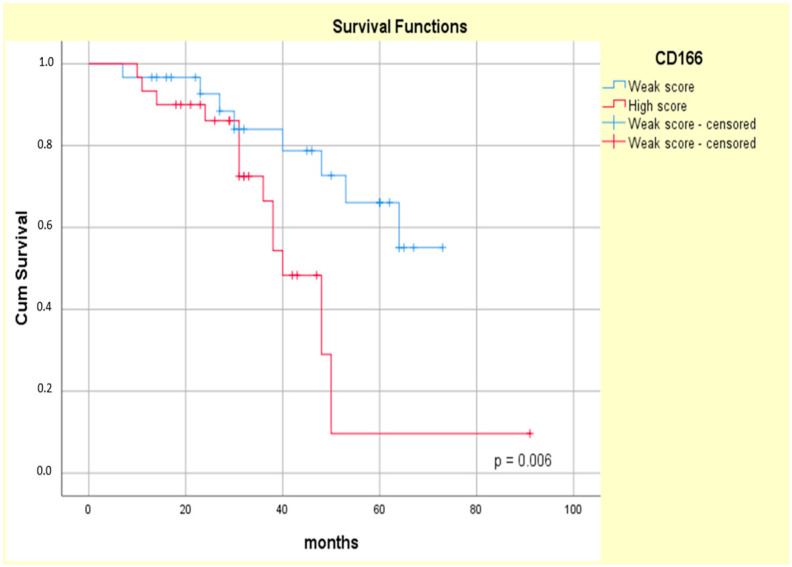
Kaplan–Meier survival curves for OS of ypT3-stage LARC patients with low or high CD166 expression.

The Kaplan–Meier curves showed that the high CD133 and CD166 expression phenotype was associated with decreased patient survival (log-rank Mantel–Cox, *p* = 0.004 and *p* = 0.006, respectively) (Figure 11 and Figure 12).

The univariate Cox regression confirmed the prognostic value of both the CD133 and CD166 markers (HR = 3.562, *p* = 0.08 and HR = 3.276, *p* = 0.010, respectively) in our study group. Additionally, high CD133 tumor expression was associated with a higher relative risk of patients’ death (HR = 3.237, *p* = 0.014) compared with high CD166 tumor expression (HR = 2.866, *p* = 0.020), as assessed by a multivariate Cox analysis (Table 6).

## 4. Discussion

The multimodal therapeutic strategy, consisting of nCRT followed by total TME, is largely accepted as the standard of care for LARC patients [15,39,40]. Accordingly, all patients in our study group had received neoadjuvant therapy, consisting of an extended course of radiation combined with concurrent chemotherapy. The radiation therapy spanned 5 weeks, delivering a total of 45 Gy in 25 fractions, followed by a supplementary dose of 5.4 Gy targeting the primary tumor. Concurrent chemotherapy was administered using CAP monotherapy, CAPEOX or FOLFOX regimens, according to the physician’s recommendation. Surgical intervention, consisting of TME, anterior resection, abdominoperineal resection or the Hartmann procedure, was performed at 6–8 weeks following the completion of the neoadjuvant therapy.

Taking into account the heterogeneity in the response to neoadjuvant therapy, variable evaluation methods have been proposed, such as tumor downstaging and the TRG system, each of them having different limitations [14,41]. Notably, RC’s response to neoadjuvant therapy may be evaluated according to the fragmentation or shrinkage pattern [14,42]. The fragmentation pattern, characterized by the main tumor’s disintegration into multiple fragments of variable shapes and sizes, is registered in about 40% of LARC cases and it is associated with minimal downstaging [9,11]. According to literature reports, ypT3-stage tumors, with residual tumor cells identified in the perirectal tissue beyond the *muscularis propria* layer [15], exhibit a tumor fragmentation pattern following nCRT [43], with a moderate or minimal therapy response in most cases and maintenance of the same diagnosis stage [15,16]. ypT3 patients who display tumor fragmentation patterns represent a subgroup of particular interest due to their high rates of RC, their poor nCRT responses and their uncertain prognoses [15,16].

One of the main problems in terms of LARC patients’ therapy responses and survival is resistance to chemoradiotherapy protocols, largely attributed to CSCs [25,38]. CRC CSCs express a panel of biomarkers, including CD24, CD26, CD29, CD133 and CD166, and have a significant role in CRC progression and metastasis [25,44,45,46,47]. CD133, a well-recognized stem cell biomarker, has been isolated in various tumors, including CRC, being considered a predictive factor associated with resistance to chemotherapy and radiotherapy [25,46,48,49]. In addition to CD133, CD166 is another well-known CSC biomarker [47,50,51]. Although some studies have found correlations between CD166 expression and a worse CRC prognosis, its significance is controversial [52,53].

Considering CSCs’ role in therapy resistance, we have evaluated the potential correlations between the clinicopathological parameters of LARC patients with a tumor fragmentation pattern following nCRT and TME and the immunoexpression of their biomarkers, aiming to refine the ypT3 stage through the identification of factors for risk stratification. A double evaluation of the CD133 and CD166 biomarkers has been used to address the limitations of previous reports of possible CD133 loss in CSCs during immunohistochemistry [44].

Most patients in our study group presented with an advanced cT stage, aligning with the findings of previous studies demonstrating that the fragmentation pattern is closely associated with advanced stages [14]. Among the different microscopy findings, LVI and PnI have been demonstrated as significant pathological features associated with local recurrence, a higher risk of metastasis and poor prognosis in RC patients following nCRT [54,55]. In this context, a recent study reported positive LVI in 7.9% of cases and positive PnI in 18.8% of LARCs with different TRGs, in patients treated with nCRT [56]. Positive LVI was registered in 51.7% of patients and positive PnI in 33.3% of cases in our study group. The registered differences between the literature data and our study results may be related to the homogeneous structure of our study group, consisting of only ypT3 cases due to the study design. Literature reports show that one-third of LARC patients achieve TRG0 post-nCRT, with most of them having an intermediate TRG response [57], which is analogous to the results obtained in our study, with only 8.3% of patients showing a TRG3 response.

Bd and PDC are also considered valuable histological prognostic features in LARC patients, being currently recommended to be constantly reported [58,59,60]. In this regard, most cases in our study group exhibited Bd1 (81.7%) and PDC1 (91.7% of cases), showing a positive response to nCRT. However, these results should be interpreted in the context of the homogeneity of the subjects’ staging and the difficulty in evaluating these parameters in the context of local fibrosis and tumor necrosis following radiotherapy. Taking these criteria into consideration, a careful evaluation of Bd, along with PDC, following neoadjuvant therapy can be helpful in risk stratification and may aid in RC patients’ clinical management [61,62,63]. Moreover, the predictive role of Bd, PDC and other negative pathological features for LARC patients’ prognosis post-nCRT needs to be further explored [59,64].

The results regarding the CD133 and CD166 immunoreactivity patterns obtained from the qualitative and semi-quantitative analyses in our study are in agreement with those of other CRC studies [7,38,65]. In particular, our results revealed a heterogeneous CD133 pattern, attributed to the variable staining intensity and to differences in subcellular localization, such as membrane and cytoplasmic staining or exclusively membrane staining [38,65]. We also registered heterogeneous CD166 immunostaining, regarding the cellular localization and intensity, exhibiting high consistency with the literature [27].

With regard to the clinicopathological parameters, our data are in agreement with those of other studies, supporting the concept that high CD133 and CD166 expression is associated with aggressive pathological features in CRC [49,66]. In particular, high CD133 expression is correlated with aggressive histopathological features, including LVI, PnI, high tumor grading and a high number of positive lymph nodes post-nCRT (*p* = 0.009, *p* = 0.006, *p* = 0.047 and *p* = 0.018, respectively), consistent with other studies’ results [7,19]. For example, a study on LARC patients following nCRT reported a higher incidence of vascular invasion in patients with high CD133 tumor immunoexpression (*p* = 0.013) [7]. Similarly, a recent study performed on young LARC patients (≤40 years) demonstrated that these tumors often exhibit aggressive pathological features, such as a higher number of lymph node metastases (*p* = 0.008) and PnI (*p* < 0.001), while they also exhibit a higher burden of CD133+ CSCs, in correlation with a poor prognosis [19]. Moreover, our study could not identify any association between CD133 tumor immunoexpression and patients’ age or gender (*p* = 0.026 and *p* = 0.592, respectively), which is in line with similar reports regarding the lack of a significant correlation between CD133 expression and CRC patients’ age (*p* = 0.267), gender (*p* = 0.93) or tumor location (*p* = 0.182) [18,25]. Taken together, this evidence suggests that high CD133 expression serves as a valuable predictor of clinically aggressive CRC and early treatment failure [18,67].

CD166’s significance in CRC is still controversial, with conflicting data reported by different research teams [30,53] (Table 7). Some studies support that low CD166 expression is associated with disease progression and poor clinical outcomes [53,68], while other studies state that high CD166 immunoexpression represents a marker of poor CRC prognosis [27,29]. In this respect, low CD166 expression was associated with higher pT (*p* = 0.014), an infiltrating growth pattern (*p* = 0.002) and reduced survival (*p* = 0.019) in a study performed on 1420 primary CRC cases [68]. However, a significant association between poor disease-free survival and high CD166 expression (*p* = 0.003) has been registered in a study performed on 112 CRC patients, suggesting that CD166 may be considered an independent prognostic marker in CRC [29]. Moreover, this observation is supported by the direct association between high CD166 expression and tumor progression and aggressive behavior (*p* = 0.01) registered in a cohort of 405 CRC cases [27]. In a contradictory manner, high CD166 expression has been associated with significantly longer OS and disease-free survival compared to cases exhibiting lower CD166 expression (*p* = 0.040 vs. *p* = 0.044), as seen in another study on 94 CRC patients [53].

In this context, our study results are in line with some previously reported data [69] regarding the correlation between CD166 expression and clinicopathological parameters, considering that a high CD166 score was statistically correlated with unfavorable clinicopathological parameters, such as PnI (*p* = 0.028). Furthermore, we observed a significant correlation between high CD166 scores and LVI (*p* = 0.020), consistent with the results of another study [70]. Thus, our research suggests that high CD166 expression may be a predictor of tumor aggressiveness and metastasis development.

An extensive analysis performed on 2048 CRC patients has demonstrated an association between high CD166 expression and a higher patient age, along with a poor degree of tumor differentiation (HR = 1.29, 95%CI = 1.01–1.29, *p* = 0.05) [30] (Table 7). However, a lack of association between CD166 expression and patients’ gender was observed, even though lower CD166 expression was noticed in male patients (HR = 0.94, 95%CI = 0.69–1.29, *p* = 0.72) [30]. This finding is in partial agreement with our study, which also noted the absence of a significant association between CD166 tumor expression and patients’ gender (*p* = 0.754). Additionally, the relatively equal distribution of male patients’ tumors exhibiting low vs. high CD166 tumor expression (38.3% vs. 40%, respectively) was most probably related to the limited number of investigated cases and the gender distribution in our study group, which was due to the accessibility of patients during the period of investigation.

In addition, our results showed that high CD166 expression was not associated with the cT stage (*p* = 0.499). Although this finding is consistent with several reports [27,70,71], other studies have found a significant association between CD166 expression and the cT stage [28,30,57,68,72,73] (Table 7). These data should be interpreted in the specific context of our study group, which was limited to ypT3 LARC patients. Other potential contributors to the contradictory reports may be represented by the different study designs, the variable structure of the study groups (with or without therapy) and the variability in the CD166 immunohistochemical scoring systems. Consequently, extensive studies on a larger cohort of patients may offer new findings to support these results.

Numerous studies have also proposed CD166 as a CSC marker in association with other markers, such as CD44 or EpCAM [27,74,75]. In a recent study, the high co-expression of EpCAM/CD166 was associated with an advanced CRC T stage, PnI and lymph node invasion [27]. In contrast, Lugli et al. found that a CD44/CD166 co-expression loss, rather than its increase, was linked to aggressive tumor-related features, including vascular invasion and advanced pT and pN stages [68]. However, further studies are needed to investigate the invasive features of CD166+ CSCs, such as LVI and PnI, to confirm that high CD166 expression could serve as a predictive factor for tumor metastasis in ypT3 LARC patients.

**Table 7:** The associations between different clinicopathological parameters and CD166 expression in relevant studies.

High CD166 Expression			
Associated Clinicopathological Features	Non-Associated Clinicopathological Features	No. of Cases	Reference
tumor progression aggressive behavior PnI lymph node invasion	cT stage	405	[27]
reduced OS cT stage	-	299	[28]
reduced OS	cT stage	110	[71]
longer OS longer disease-free survival	-	94	[53]
patients’ age poor degree of tumor differentiation cT stage	patients’ gender	2048 *	[30]
cT stage	-	1521 *	[57]
		1420	[68]
		45	[72]
		120	[73]
-	patients’ gender lymph node status cT stage	3332	[70]
reduced disease-free survival	-	112	[29]
**Low CD166 expression**			
higher pT stage infiltrating growth pattern reduced survival pN stage	-	1420	[68]

cT stage—clinical tumor stage; PnI—perineural invasion; pT—pathological tumor; pN—pathological lymph node; OS—overall survival; *—meta-analysis.

Tumor Bd, possibly composed of a migratory population of CSCs [76], represents a feature of poor CRC prognosis, being largely associated with poor tumor differentiation and lymph node metastases [77]. Although considered a CSC niche, Bd tumor cells may express different biomarkers compared to the main tumor [78,79]. While CD44, E-cadherin, CD166 and EpCAM were expressed in the medial membranes of tumor cells, they were not expressed in the tumor buddings in a recent study [78]. CD90, CD133 and CD44s expression was detected in less than 5% of tumor buds, while CD166, CD24 and ALDH1 were expressed in 34%, 16.2% and 16.5% of cases, respectively, in a study evaluating eight CSC biomarkers [79]. Moreover, according to the same study, unlike CD166, CD133, CD24, CD90, CD44s and ALDH1, the expression of EpCAM and ABCG5 within tumor buds is often associated with a poor prognosis [79]. Notably, our study did not find any significant association between high CD166 expression and Bd, which was probably related to the homogenous Bd pattern in the study group, with individual tumor cells or small clusters identified at the ITF, evaluated as Bd1 in most of the investigated cases.

Several studies have demonstrated a direct correlation between CD133 expression and OS in various malignancies, including breast cancer, esophageal cancer, glioblastoma, osteosarcoma, hepatocellular carcinoma and CRC [67,80,81,82,83,84,85]. In agreement with these reports, we found that CD133 immunoexpression was significantly associated with the survival period in our study group (*p* = 0.004). Despite the reports of an association between CD133 expression and poor OS in CRC by some research teams [26,38,82], contradictory findings have been reported by other research teams [25,68,86,87]. For instance, a study on CRC with peritoneal metastases did not find any correlation between CD133 expression and overall patients’ survival, with longer disease-free survival in CD133-positive patients compared to CD133-negative patients [85]. Additionally, another study reported no significant correlation between CD133 expression and CRC patients’ 5-year survival (*p* = 0.054) [25].

The literature reports regarding CD166 and the survival of patients diagnosed with CRC are highly contradictory [88]. Some studies have reported that high CD166 expression is correlated with poor survival [30,89,90], while other studies have demonstrated its association with increased survival [28,53]. However, a recent meta-analysis reported the opposite result, supporting the notion that high CD166 expression is associated with poor CRC OS (*p* < 0.00001) [30], as we also noticed in our study (*p* = 0.006). Moreover, three studies using multivariate regression analysis could not find any significant association between CD166 expression and OS [28,74,89]. In this context, our results may be related to the study design and to the CSC population’s heterogeneity.

Although CRC CSCs may display a panel of biomarkers, their expression seems to exhibit plasticity and intrinsic fluctuations, which may contribute to the variable results of marker-based CSC assays [20,91,92]. In this regard, a heterogeneous population of CD133+ and CD133− CRC CSCs has been identified, attributed to specific mutations of the RAS-RAF axis [20,92,93].

CRC CSCs are also able to survive following therapeutic interventions; CD133+ CRC CSCs have been demonstrated as more resistant to 5-fluorouracil, with higher invasiveness and metastatic potential compared to CD133- CRC CSCs [45,92]. Additionally, increased resistance to radiotherapy has been identified in HT-29 human intestinal colon cancer lines [94].

**Figure 13:**
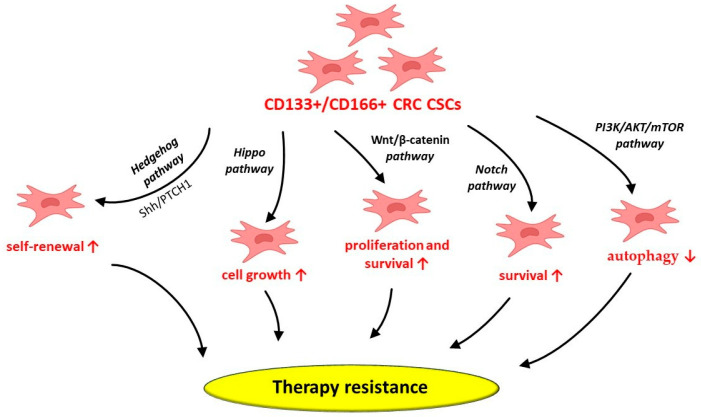
Potential mechanisms behind CD133+/CD166+ CRC CSCs’ involvement in therapy resistance. CRC—colorectal cancer; CSCs—cancer stem cells; CD—cluster of differentiation; PTCH1—transmembrane receptor PATCHED1; Shh—sonic Hedgehog protein; ↑—increase; ↓—decrease.

CRC CSCs’ ability to escape conventional therapy may be attributed to the aberrant activation of growth and survival pathways, such as Wnt/β-catenin, Hedgehog, Notch and Hippo [21] (Figure 13). Cancer cells’ growth and progression, especially in aggressive and drug-resistant tumors, may be induced by abnormal Hedgehog pathway activation [21]. In this regard, the Hedgehog-GLI (HH-GLI) pathway’s activation by the exo-secretory ligand Shh, which binds to the transmembrane receptor PATCHED1 (PTCH1), may contribute to the maintenance of CD133+ colon CSCs’ self-renewal ability [21,95]. Additionally, the existence of CRC CSCs with high Wnt signaling activity may suggest a potential therapeutic strategy by targeting the Wnt pathway in order to modulate the stem cell population niche [21]. The aberrant activation of the Notch pathway components, mainly the HES gene family, in CRC CSCs may also support CRC recurrence and therapeutic resistance [21]. Consequently, targeting Notch signaling may represent a potential strategy for CRC treatment. Moreover, the Hippo pathway’s activation has been demonstrated to modulate CRC cell growth. In this context, OPCs, usually found in vegetables and fruits, have been shown to display anti-CSC activity by inhibiting the Hippo axis [21,95,96]. As a consequence, deeper insight into the mechanisms involved in CRC CSCs’ survival may lead to novel therapeutic strategies for these patients.

CD133+ CRC CSCs may also depend on autophagy to survive in hypoxic tumor microenvironments, including during cancer therapy [97]. In this regard, CD133+ CRC CSCs suppress autophagy through PI3K/AKT/mTOR signaling pathway activation [97]. However, various factors, such as the unique cellular and molecular structure of the CRC tumor niche, along with external influences on this specific pathway, may alter the delicate balance between autophagy and cell survival mediated by CD133+ CRC CSCs [97].

As a consequence, the inhibition of their autophagy may represent a possible therapeutic tool to address CRC CSCs’ therapy resistance [97]. However, it must be acknowledged that CD133+ CSCs are a heterogeneous cell population that co-express additional cell surface receptors. Among these surface receptors, CXCR4 is able to modulate a critical paracrine signaling axis, demonstrating that the CD133+ phenotype provides enhanced tumorigenic properties [98].

These accumulated data demonstrate the large heterogeneity of CRCs, suggesting that CD133 targeting may not be effective. The better prognosis stratification of these patients may be achieved via the comprehensive immunohistochemical evaluation of residual CSCs [29,97,99]. In this context, our study showed that ypT3 LARC patients with high CD133 and CD166 expression exhibited lower OS compared to ypT3 LARC patients with low CD133 and CD166 expression. Moreover, the multivariate analysis suggested that high CD133 expression (HR = 3.237, *p* = 0.014, CI = 95%) is an independent predictive factor for reduced OS, with a more significant impact on survival in comparison to high CD166 expression (HR = 2.866, *p* = 0.020, CI = 95%). Additionally, in selected cases showing CD133 negativity, residual CSCs may be highlighted by adding CD166 immunohistochemistry, which may provide an improved picture of ypT3 LARC patients’ post-therapeutic response. Lastly, CD166 may be considered a suitable indicator for patients’ drug treatment responses in some cancer types [51].

To our knowledge, this is the first report of CD133/CD166 double expression in LARC patients diagnosed with ypT3 and exhibiting a tumor fragmentation response to nCRT. However, our preliminary study had some limitations attributed to the relatively reduced number of cases included in the study, necessitating future validation via a supplementary statistical analysis in a larger cohort of ypT3-stage post-nCRT LARC patients. Additionally, the use of several CRC CSC markers in addition to CD133 and CD166 would contribute to a better assessment of the post-therapeutic status of these patients and, thus, to therapy and prognosis stratification. Moreover, performing large studies validated by multiple comparative statistical analyses on large cohorts of patients with variable ypT stages after therapy could provide a reliable picture of the therapeutic response of these patients and may enable an understanding of the CRC CSCs’ avoidance mechanisms under current therapies.

Nonetheless, the partial agreement of our results with those of other studies may be primarily attributed to differences in the study design, as well as the histochemical properties of each biomarker and the different clinicopathological parameters evaluated. In agreement with different published immunohistochemical and molecular studies on a limited number of cases [53,66,72,100,101], our results highlight the complex behavior of CSCs in LARC patients treated with nCRT.

## 5. Conclusions

Our study shows that LARC is characterized by a complex and heterogeneous histopathological profile, in agreement with the literature stating that the ypT3 stage is a challenging entity, with debatable prognostic significance. The response pattern to nCRT, as a relatively new parameter, seems to have potential predictive value, and our study supports its inclusion in the histopathological evaluation of resection specimens.

In addition, our findings highlight the possible correlations between CD133 and CD166 expression and the clinicopathological characteristics, which must be confirmed by future studies performed in larger, independent cohorts of ypT3 LARC patients. These correlations are suggestive of aggressive tumor behavior, highlighting their potential value as prognostic factors.

## Figures and Tables

**Table 1:** Dworak tumor response grading system (TRG).

TRG Grade	Response	Regression Characteristics
0	no response	no regression of tumor mass
1	minimal response	evident tumor mass with prominent fibrosis and/or vasculopathy
2	moderate response	predominantly fibrotic changes, with a few easily identifiable tumor cells or groups
3	near-complete response	rare identifiable tumor cells within the fibrotic tissue, with or without mucin
4	complete response	total regression of tumor mass, without any identifiable tumor cells

**Table 2:** CD133 expression scoring system.

Staining Pattern	Score	Category
no staining	0	low expression
<50% luminal staining	1	
<50% luminal and cytoplasmic staining	2	
≥50% luminal staining	3	high expression
≥50% luminal and cytoplasmic staining	4	

**Table 3:** CD166 expression scoring system.

Membrane Staining Intensity	Score	Percentage of Positive Cells	Score	Category
negative	0	0–100%	staining intensity score x percentage of positive cells (cut-off value = 120)	low expression (≤120)
weak	1			
moderate	2			high expression (>120)
strong	3			

**Table 4:** Clinical and pathological characteristics of ypT3-stage LARC patients.

Clinicopathological Characteristics		*n* (%)
**Age**	45–55 years	12 (20%)
	56–65 years	21 (35%)
	66–75 years	23 (38.3%)
	76–85 years	4 (6.7%)
**Gender**	women	13 (21.7%)
	men	47 (78.3%)
**cT stage**	T2	3 (5.0%)
	T3	44 (73.3%)
	T4	13 (21.7%)
**cN stage**	N0	2 (3.3%)
	N1	7 (11.7%)
	N2	43 (71.7%)
	Nx	8 (13.3%)
**ypN category**	ypN0	32 (53.3%)
	ypN1	21 (35%)
	ypN2	7 (11.7%)
**Histological type**	AC NOS	54 (90%)
	mucinous AC	6 (10%)
**Grading**	low	47 (78.3%)
	high	13 (21.7%)
**TRG**	TRG 0	2 (3.3%)
	TRG 1	25 (41.7%)
	TRG 2	28 (46.7%)
	TRG 3	5 (8.3%)
**LVI**	negative	29 (48.3%)
	positive	31 (51.7%)
**IMVI**	negative	29 (48.3%)
	positive	31 (51.7%)
**EMVI**	negative	39 (65%)
	positive	21 (35%)
**PnI**	negative	40 (66.6%)
	positive	20 (33.4%)
**Bd**	Bd 1	49 (81.6%)
	Bd 2	7 (11.7%)
	Bd 3	4 (6.7%)
**PDC**	PDC1	55 (91.7%)
	PDC2	4 (6.7%)
	PDC3	1 (1.6%)
**nCRT**	CAP-RT	50 (83.4%)
	CAPEOX-RT	8 (13.3%)
	FOLFOX-RT	2 (3.3%)
**Type of surgical procedure**	anterior resection and TME	30 (50%)
	abdominoperineal resection	25 (41.7%)
	Hartmann procedure and TME	5 (8.3%)
**Status**	survivor	37 (61.7%)
	non-survivor	23 (38.3%)
**Mean OS ± SD (months)** **Female** **Male**	37.48 ± 18.06	
	36.15 ± 22.63	-
	37.85 ± 16.86	

AC—adenocarcinoma; Bd—tumor budding; CAP-RT—capecitabine and radiotherapy; CAPEOX-RT—capecitabine, oxaliplatin and radiotherapy; cN stage—clinical N stage; cT stage—clinical T stage; EMVI—extramural vascular invasion; FOLFOX-RT—leucovorin calcium, fluorouracil and oxaliplatin and radiotherapy; IMVI—intramural vascular invasion; LVI—lymphovascular invasion; N—regional lymph nodes; nCRT—neoadjuvant chemoradiotherapy; NOS—not otherwise specified; OS—overall survival; PDC—poorly differentiated clusters; PnI—perineural invasion; T—tumor; TME—total mesorectal excision; TRG—tumor regression grade; ypN—lymph node status after neoadjuvant therapy.

**Table 5:** Relationships between CD133 and CD166 scoring and clinicopathological characteristics of ypT3-stage LARC patients.

Clinicopathological Parameter	CD133 Expression		*p*-Value	CD166 Expression		*p*-Value
	Low (*n*; %)	High (*n*; %)		Low (*n*; %)	High (*n*; %)	
**Age**						
45–55 years	6 (10%)	6 (10%)	*p* = 0.260	5 (8.3%)	7 (11.7%)	*p* = 0.121
56–65 years	10 (16.6%)	11 (18.3%)		14 (23.3%)	7 (11.7%)	
66–75 years	16 (26.7%)	7 (11.7%)		8 (13.3%)	15 (25%)	
76–85 years	1 (1.6%)	3 (5%)		3 (5%)	1 (1.6%)	
**Gender**						
Female	8 (13.3%)	5 (8.3%)	*p* = 0.592	7 (11.7%)	6 (10%)	*p* = 0.754
Male	25 (41.7%)	22 (36.7%)		23 (38.3%)	24 (40%)	
**cT stage**						
T2	3 (5%)	-	*p* = 0.054	1 (1.6%)	2 (3.3%)	*p* = 0.499
T3	26 (43.3%)	18 (30%)		24 (40%)	20 (33.4%)	
T4	4 (6.7%)	9 (15%)		5 (8.3%)	8 (13.3%)	
**cN stage**						
N0	2 (3.3%)	-	*p* = 0.330	1 (1.6%)	1 (1.6%)	*p* = 0.219
N1	5 (8.3%)	2 (3.3%)		5 (8.3%)	2 (3.3%)	
N2	23 (38.3%)	20 (33.4%)		18 (30%)	25 (41.7%)	
Nx	3 (5%)	5 (8.3%)		6 (10%)	2 (3.3%)	
**ypN category**						
yN0	23 (38.3%)	9 (15%)	*p* = 0.018	19 (31.7%)	13 (21.7%)	*p* = 0.242
yN1	7 (11.7%)	14 (23.3%)		9 (15%)	12 (20%)	
yN2	3 (5%)	4 (6.7%)		2 (3.3%)	5 (8.3%)	
**Histological type**						
AC NOS	31 (51.7%)	23 (38.3%)	*p* = 0.261	28 (46.7%)	26 (43.3%)	*p* = 0.389
mucinous AC	2 (3.3%)	4 (6.7%)		2 (3.3%)	4 (6.7%)	
**Grading**						
low grade	29 (48.3%)	18 (30%)	*p* = 0.047	26 (43.3%)	21 (35%)	*p* = 0.117
high grade	4 (6.7%)	9 (15%)		4 (6.7%)	9 (15%)	
**TRG**						
TRG 0	1 (1.6%)	1 (1.6%)	*p* = 0.079	1 (1.6%)	1 (1.6%)	*p* = 0.873
TRG 1	9 (15%)	16 (26.7%)		14 (23.3%)	11 (18.3%)	
TRG 2	19 (31.7%)	9 (15%)		13 (21.7%)	15 (25%)	
TRG 3	4 (6.7%)	1 (1.6%)		2 (3.3%)	3 (5%)	
**LVI**						
Negative	21 (35%)	8 (13.3%)	*p* = 0.009	19 (31.7%)	10 (16.6%)	*p* = 0.020
Positive	12 (20%)	19 (31.7%)		11 (18.3%)	20 (33.4%)	
**IMVI**						
Negative	17 (28.3%)	12 (20%)	*p* = 0.586	18 (30%)	11 (18.3%)	*p* = 0.071
Positive	16 (26.7%)	15 (25%)		12 (20%)	19 (31.7%)	
**EMVI**						
Negative	25 (41.7%)	14 (23.3%)	*p* = 0.053	16 (26.7%)	23 (38.3%)	*p* = 0.058
Positive	8 (13.3%)	13 (21.7%)		14 (23.3%)	7 (11.7%)	
**PnI**						
Negative	27 (45%)	13 (21.7%)	*p* = 0.006	24 (40%)	16 (26.7%)	*p* = 0.028
Positive	6 (10%)	14 (23.3%)		6 (10%)	14 (23.3%)	
**Bd**						
Bd 1	29 (48.3%)	20 (33.4%)	*p* = 0.330	26 (43.3%)	23 (38.3%)	*p* = 0.515
Bd 2	3 (5%)	4 (6.7%)		3 (5%)	4 (6.7%)	
Bd 3	1 (1.6%)	3 (5%)		1 (1.6%)	3 (5%)	
**PDC**						
**PDC 1**	31 (51.7%)	24 (40%)	*p* = 0.521	28 (46.7%)	27 (45%)	*p* = 0.601
**PDC 2**	2 (3.3%)	2 (3.3%)		2 (3.3%)	2 (3.3%)	
**PDC 3**	-	1 (1.6%)		-	1 (1.6%)	

Pearson’s χ2 test—significant *p*-value < 0.05; AC—adenocarcinoma; Bd—tumor budding; cN stage—clinical N stage; cT stage—clinical T stage; EMVI—extramural vascular invasion, IMVI—intramural vascular invasion, LVI—lymphovascular invasion; N—regional lymph nodes; nCRT—neoadjuvant chemoradiotherapy; NOS—not otherwise specified; OS—overall survival; PDC—poorly differentiated clusters; PnI—perineural invasion; T—tumor; TME—total mesorectal excision; TRG—tumor regression grade; ypN—lymph node status after neoadjuvant therapy.

**Table 6:** Cox regression analysis of prognostic value of CD133 and CD166 tumor expression in ypT3-stage LARC patients.

	OS			
	Univariate Analysis		Multivariate Analysis	
	HR (95% CI)	*p*-Value	HR (95% CI)	*p*-Value
CD133 expression	3.562 (1.397–9.082)	0.008	3.237 (1.268–8.263)	0.014
CD166 expression	3.276 (1.325–8.102)	0.010	2.866 (1.184–6.940)	0.020

CI—confidence interval, HR—hazard ratio, OS—overall survival; significant *p*-value < 0.05.

## Data Availability

The original contributions presented in this study are included in the article. Further inquiries can be directed to the corresponding authors.

## Abbreviations

The following abbreviations are used in this manuscript:

ABCG5	ATP-binding cassette subfamily G member 5
ALDH1	Aldehyde dehydrogenase 1
AJCC	American Joint Committee on Cancer
Bd	Tumor budding
CAP	Capecitabine
CAPEOX	Capecitabine and oxaliplatin
CD44	Cluster of Differentiation 44
CRC	Colorectal cancer
CRM	Circumferential resection margin
CSCs	Cancer stem cells
CXCR4	C-X-C chemokine receptor type 4
EMVI	Extramural vascular invasion
EpCAM	Epithelial cell adhesion molecules
FOLFOX	Leucovorin calcium, fluorouracil and oxaliplatin
IMVI	Intramural vascular invasion
ITBCC	Tumor Budding Consensus Conference
LARC	Locally advanced rectal cancer
ITF	Invasive tumor front
LVI	Lymphovascular invasion
nCRT	Neoadjuvant chemoradiotherapy
OPCs	Oligomeric proanthocyanidins
OS	Overall survival
PTCH1	Transmembrane receptor PATCHED1
PDCs	Poorly differentiated clusters
PnI	Perineural invasion
RC	Rectal cancer
TME	Total mesorectal excision
TRG	Tumor regression grade
ypN	Lymph node status after neoadjuvant therapy
